# Incomplete immunization among children aged 12–23 months in Togo: a multilevel analysis of individual and contextual factors

**DOI:** 10.1186/s12889-018-5881-z

**Published:** 2018-08-02

**Authors:** Didier K. Ekouevi, Fifonsi A. Gbeasor-Komlanvi, Issifou Yaya, Wendpouiré I. Zida-Compaore, Amevegbé Boko, Essèboe Sewu, Anani Lacle, Nicolas Ndibu, Yaovi Toke, Dadja E. Landoh

**Affiliations:** 1 Département de Santé Publique, Université de Lomé, Faculté des Sciences de la Santé, Lomé, Togo; 2 Centre Africain de Recherche en Epidémiologie et en Santé Publique (CARESP), Lomé, Togo; 3 ISPED, Université de Bordeaux & Centre INSERM U1219 - Bordeaux Population Health, Bordeaux, France; 4 Sciences Economiques & Sociales de la Santé & Traitement de l’Information Médicale (SESSTIM), Aix Marseille Université, Marseille, France; 5 Programme Elargi de Vaccination, Ministère de la Santé et de la Protection Sociale, Lomé, Togo; 6 UNICEF, country office of Togo, Lomé, Togo; 7 World Health Organization, country office of Togo, Lomé, Togo

**Keywords:** Incomplete immunization coverage, Children, Obstacles, Associated factors, Togo

## Abstract

**Background:**

Inadequate immunization coverage remains a public health problem in Africa. In Togo, only 62% of children under one year of age were fully immunized in 2013. This study aimed to estimate the immunization coverage among children aged 12–23 months, and to identify factors associated with incomplete immunization status in Togo.

**Methods:**

A cross-sectional survey was conducted in the six health regions of Togo. Children aged 12 to 23 months who were living with one of their parents or guardians from selected households were recruited for the study. Data was collected using a pre-tested questionnaire through face-to-face interviews. Multilevel logistic regression analyses were performed to assess factors associated with incomplete immunization coverage.

**Results:**

A total of 1261 households were included. Respondents were predominantly women (91.9%) and 22.8% had secondary or higher education level. Immunization cards were available for 85.3% of children. Complete immunization coverage was 72.3%, 95% confidence interval (CI): [69.7–74.8]). After controlling for both individual and contextual level variables, children whose mothers attended secondary school or above were 33% (adjusted Odds Ratio (aOR) = 0.67, CI [0.47–0.94]) less likely to have an incomplete immunization coverage compared to those with no education. The likelihood of incomplete immunization in children decreased with the increase in household’s income (aOR = 0.73, 95% CI [0.58–0.93]), children who did not have an immunization card (aOR = 13.41, 95% CI [9.19–19.57]) and those whose parents did not know that children immunization was free of charge (aOR = 1.82, 95% CI [1.00–3.30]) were more likely to have an incomplete immunization. Finally, children whose parents had to walk half an hour to one hour to reach a healthcare center were 57% (aOR = 1.57, 95% CI [1.15–2.13]) more likely to have an incomplete immunization coverage than those whose parents had to walk less than half an hour.

**Conclusion:**

The goal of 90% coverage at the national level has not been achieved in 2017. Innovative strategies such as using electronic cards and strengthening sensitization activities must be initiated in order to attain a complete immunization coverage in Togo.

## Background

Complete immunization of children under one year of age remains one of the most cost-effective strategies to reduce child mortality and to help achieve Sustainable Development Goals (SDG) [1]. With children’s immunization, 2 to 3 million deaths from diphtheria, tetanus, pertussis, and measles are avoided each year [1, 2]. Hence, every child should benefit equitably from the administration of all routine vaccines and the World Health Organization (WHO) recommends that complete immunization coverage should reach at least 90% of children at country level and 80% at district level [2, 3]. However, some children are completely immunized while others are not, leading to a disparity in immunization coverage [4–6]. Although global coverage has increased since the implementation of the Expanded Program on Immunization (EPI), in 2016, only 86% of children worldwide received 3 doses of vaccine containing diphtheria-tetanus-pertussis (DTP3) during their first year of life. In sub-Saharan Africa countries, global immunization coverage remains low and vaccine-preventable diseases constitute a major contributor to high child mortality rates [7, 8]. The reasons for incomplete immunization among children have been grouped into three categories: reasons relating to the parents, those relating to healthcare system and healthcare providers [4, 5, 9–11].

In Togo, the EPI has been implemented by health authorities since 1980. It currently includes seven vaccines that are recommended for children under one year of age: the tuberculosis vaccine (BCG); the pentavalent vaccine (PENTA) against diphtheria, tetanus, pertussis, hepatitis B and invasive Haemophilus influenzae type b infections; the poliomyelitis vaccine (referred to as OPV for the oral form); the pneumococcal vaccine (PNEUMO); the vaccine against rotavirus gastroenteritis (ROTA); the measles vaccine and the yellow fever vaccine. These vaccines are administered during five immunization sessions at birth, at 6, 10, 14 weeks and 9 months of age. Based on data from Togo’s fourth Multiple Indicator Cluster Survey (MICS4) in 2010, Landoh et al. [12] showed that almost 36.2% of children aged one to five years had not received all vaccines recommended by the EPI. Also in the third Demographic and Health Survey (DHS) report in Togo in 2013 [13], about 40% of the children under one year of age were not completely immunized according to the immunization schedule.

Despite the implementation of outreach strategies and supplementary immunization activities, full immunization coverage remains below target in Togo. Therefore, it appears necessary to conduct operational research to identify barriers to complete immunization coverage as well as factors associated with the disparity in the immunization coverage. The objective of this study was to estimate the immunization coverage among children aged 12 to 23 months and identify factors associated with incomplete immunization coverage in Togo in 2017.

## Methods

### Study design and sampling

A descriptive and analytical cross-sectional study was carried out from February 27th to March 5th 2017 in the six health regions (HR) of Togo: Lomé, Maritime, Plateaux, Centrale, Kara, and Savanes. The World Health Organization (WHO) cluster survey methodology was used for the immunization coverage survey [14]. The sampling frame consisted of all the neighborhoods / villages / avenues of each selected prefecture. Thus, 30 clusters per health region were randomly selected and distributed by prefecture and canton. In each cluster, seven households were chosen by lot and interviewed. Overall, for the six health regions in Togo, the sample should include at least 1260 households. Therefore, the household was used as the main sampling unit.

All children aged 12–23 months living in selected households and with one of their parents or guardians were included in the study. A sample of 1128 children aged 12–23 months was calculated using a single proportion population formula with a 95% confidence level, 7% margin of error and 60% estimated immunization coverage rate in the study area [13]. A 10% non-response rate was considered and the minimum number of respondent-child couples required in each health region was estimated at 207.

### Data collection

A pre-tested quantitative questionnaire was administered during a face-to-face interview to the person who usually takes the child to the immunization center. The questionnaire included questions on socio-demographic characteristics of the respondent and the eligible child, possession of a vaccination card for the child, vaccines administered to the child (based on immunization records or respondent’s declarative statements), knowledge, attitudes and practices regarding immunization of children, and accessibility to immunization services. Data collection was carried out by investigators who participated in a two-day training session in order to master data collection tools and techniques for locating survey areas. All data collection forms completed by the interviewers were double checked by study supervisors for completeness and consistency. Validated forms were then sent to the EPI department for data entry.

### Variables of the study

#### Outcome variable

Immunization status of children. Since our study focused on children aged between 12 and 23 months, the definition of vaccination coverage was based on the immunization schedule adopted by the EPI in Togo and the data available on immunization card or recall of parents/guardians. Therefore, children were classified into three categories: “fully immunized child” who had received all the routine vaccines recommended by EPI before turning one year old; “partially immunized child” who missed at least any one dose of the routine vaccines; “unimmunized child” who had not received any vaccine by 12 months of age.

### Statistical analysis

Data entry and cleaning were carried out by a team of ten data entry operators, a supervisor and a data transcriber previously trained and under the supervision of a database manager. A database was designed with the Epidata software version 3.1. Descriptive analyses were performed and results were presented as proportions (% of children with complete immunization) with their 95% confidence interval. Comparisons of the qualitative variables were performed using chi-square or Fisher’s exact tests and comparisons of medians were performed using nonparametric Kruskall Wallis or Wilcoxon tests. Univariate and multivariate multilevel logistic regression analyses were performed to study the relationship between the dependent variable ‘incomplete immunization coverage’ and the individual and contextual variables. The dependent variable ‘incomplete immunization coverage’ was coded as 0 for fully immunized children and 1 for unimmunized or partially immunized children. The explanatory variables were presented into two categories for regression analyses: individual factors (socio-demographic characteristics of the parents/guardians, knowledge of mothers/guardians that vaccine is free of charge, possession of immunization cards) and contextual factors (walking time to immunization centers and health regions). All analyses were carried out using STATA® software version 11.0 (StataCorp, College Station, Texas, USA).

### Ethical consideration

This study was approved by the National Ethics Committee of the Ministry of Health in Togo and all respondents signed an informed consent form before enrolment in the study.

## Results

Of 1262 households identified, 1261 were enrolled in the six health regions in Togo, with 210 or 211 households per health region. It should be noted that in one household, one parent did not give consent for the survey, yielding a response rate of 99.92%.

### Sociodemographic characteristics of parents or guardians of children aged 12 to 23 months

Respondents in households were predominantly female (91.91%), married (93.42%) and only 22.76% had secondary or higher education. In most households (94.69%) there was only one child aged 12–23 months. In the Savanes-HR, 18.1% of parents or guardians reported having more than one child aged 12–23 months in their household compared with 1.90% in Lomé-HR and Centrale-HR, 2.39% in Kara-HR, and 2.80% in Plateaux-HR and Maritime-HR. Respondents’ sociodemographic characteristics and household characteristics are presented in Table 1.

**Table 1:** Characteristics of the parents or guardians

Baseline characteristics	Health region												TOTAL	
	Lomé (*n* = 211)		Maritime (*n* = 210)		Plateaux (*n* = 210)		Centrale (*n* = 211)		Kara (*n* = 209)		Savanes (*n* = 210)		*N* = 1261	
	N	%	n	%	n	%	n	%	n	%	n	%	N	%
Age at enrolment (years)														
< 15	1	0.47	0	0.00	3	1.43	3	1.42	3	1.44	1	0.48	11	0.87
15–49	205	97.16	199	94.76	198	94.29	197	93.36	189	90.43	205	97.62	1193	94.61
> 49	4	1.90	4	1.91	3	1.43	7	3.32	11	5.26	0	0.00	29	2.30
Missing data	1	0.47	7	3.33	6	2.85	4	1.90	6	2.87	4	1.90	28	2.22
Education level														
No education	60	28.44	60	28.57	56	26.67	47	22.27	102	48.80	149	70.95	474	37.59
Primary	80	37.91	100	47.62	90	42.85	104	49.29	53	25.36	24	11.43	451	35.76
Secondary	68	32.23	36	17.15	53	25.34	50	23.70	47	22.49	16	7.62	270	21.41
Higher	2	0.95	7	3.33	5	2.38	1	0.47	1	0.48	1	0.48	17	1.35
Missing data	1	0.47	7	3.33	6	2.85	9	4.27	6	2.87	20	9.52	49	3.89
Monthly income														
< 30,000 FCFA	189	89.58	194	92.38	197	93.81	194	91.94	198	94.74	207	98.57	1179	93.50
≥ 30 00 FCFA	21	9.95	14	6,67	12	5.71	16	7.58	4	1.91	3	1.43	70	5.55
Missing data	1	0.47	2	0.95	1	0.48	1	0.48	7	3.35	0	0.00	12	0.95
Walking time to reach healthcare center (minutes)														
< 30	97	45.97	101	48.09	106	50.48	59	27.96	67	32.06	45	21.43	475	37.67
30–60	113	53.56	57	27.14	57	27.14	64	30.33	55	26.31	85	40.47	431	34.18
> 60 min	1	0.47	52	24.77	44	20.95	87	41.24	86	41.15	79	37.62	349	27.68
Missing data	0	0.00	0	0.00	3	1.43	1	0.47	1	0.48	1	0.48	6	0.47
Occupation														
Not working	10	4.74	27	12.86	4	1.91	1	0.47	6	2.87	1	0.48	49	3.89
Worker	6	2.84	10	4.76	2	0.95	5	2.37	2	0.96	1	0.48	26	2.06
Farming	0	0.00	57	27.14	113	53.81	102	48.34	66	31.58	152	72.38	490	38.86
Income-generating activities	145	68.72	72	34.29	31	14.76	49	23.22	95	45.45	26	12.38	418	33.15
Other activities	43	20.38	25	11.90	46	21.90	42	19.91	38	18.18	27	12.85	221	17.52
Missing data	7	3.32	19	9.05	14	6.67	12	5.69	2	0.96	3	1.43	57	4.52
Marital status														
Never in union	4	1.90	6	2.86	3	1.43	5	2.37	6	2.87	0	0.00	24	1.90
Married	201	95.26	195	92.86	188	89.52	200	94.79	189	90.44	205	97.62	1178	93.42
Divorced	0	0.00	5	2.38	0	0.00	1	0.47	0	0.00	0	0.00	6	0.48
Widow	3	1.42	1	0.48	4	1.90	2	0.96	7	3.35	0	0.00	17	1.35
Separated	2	0.95	3	1.42	10	4.76	1	0.47	3	1.43	3	1.43	22	1.74
Missing data	1	0.47	0	0.00	5	2.39	2	0.94	4	1.91	2	0.95	14	1.11

(*MD*) Missing data

### Immunization card possession

Immunization cards were available for review for 85.33% of the respondents. There is no difference in terms of sociodemographic characteristics (age, level of education, or income) between parents or guardians of children based on immunization cards possession (*p* > 0.05). The proportion of children who had an immunization card was higher in the health regions of Lomé, Centrale and Savanes with 91.00%, 90.52% and 86.67%, respectively. The rate of non-possession of immunization card was higher in the Plateaux-HR where one in four respondents could not present an immunization card for a child under 2 years of age residing in the household. The reasons given by the participants for the absence of an immunization card were the loss of the card (32.12%), the unavailability of the card (49.70%) and the fact that the child had been vaccinated without being given a card (10.91%). The unavailability of the card in the household was mentioned at 70.59% in Lomé-HR and at 54.84% in the Maritime-HR. In the Plateaux-HR, 40% of parents or guardians of children without an immunization card had indicated the loss of the immunization card, compared with 56% in the Savanes-HR.

### Vaccines administered to children aged 12 to 23 months

Nine in ten children (90.72%) were immunized against tuberculosis. The proportion of children who received the first dose of pentavalent vaccine was 83.90%, but this proportion decreased gradually from the first dose to the third one which was 81.21%. The same trend was observed for the oral polio vaccine (OPV) with a proportion of children vaccinated dropping from 88.26% for the first dose to 86.52% for the second dose and then to 85.25% for the third dose (Table 2). Lomé-HR was the health region with the best immunization coverage. All children were immunized against tuberculosis and 99.05% received the first dose of vaccine against polio and pentavalent vaccine. Immunization coverage varied between 96.83% for yellow fever vaccine and 99.63% for BCG in children with an immunization card. In the Centrale-HR, all respondents whose children had an immunization card received BCG, three doses of oral poliomyelitis vaccine, three doses of pentavalent vaccine, three doses of pneumococcal vaccine, two doses of vaccine against Rotavirus, and vaccines against measles and yellow fever. For children without an immunization card, immunization coverage varied between 64.58% for yellow fever vaccine and 86.30% for BCG.

**Table 2:** Immunization coverage by region among children aged 12–23 months

	Health region													
	Lomé		Maritime		Plateaux		Centrale		Kara		Savanes		Total	
	(n = 211)		(n = 210)		(n = 210)		(n = 211)		(n = 210)		(n = 210)		(n = 1261)	
	n	%	n	%	n	%	n	%	n	%	N	%	n	%
BCG	211	100	206	98.10	160	76.19	210	99.53	173	82.38	184	87.62	1144	90.72
OPV-1	209	99.05	178	84.76	160	76.19	210	99.53	172	81.90	184	87.62	1113	88.26
OPV-2	206	97.63	168	80.00	156	74.29	210	99.53	171	81.43	180	85.71	1091	86.52
OPV-3	199	94.31	165	78.57	155	73.81	210	99.53	168	80.00	178	84.76	1075	85.25
PENTA-1	209	99.05	182	86.67	105	50	210	99.53	168	80.00	184	87.62	1058	83.90
PENTA-2	206	97.63	175	83.33	104	49.52	210	99.53	165	78.57	181	86.19	1041	82.55
PENTA-3	199	94.31	169	80.48	106	50.48	210	99.53	162	77.14	178	84.76	1024	81.21
PNEUMO-1	208	98.58	180	85.71	158	75.24	207	98.1	168	80.00	183	87.14	1104	87.55
PNEUMO-2	205	97.16	174	82.86	156	74.29	207	98.1	165	78.57	179	85.24	1086	86.12
PNEUMO-3	199	94.31	167	79.52	155	73.81	210	99.53	162	77.14	177	84.29	1070	84.85
ROTA-1	207	98.1	176	83.81	161	76.67	210	99.53	167	79.52	183	87.14	1104	87.55
ROTA-2	204	96.68	173	82.38	159	75.71	209	99.05	165	78.57	181	86.19	1091	86.52
Measles	179	84.83	156	74.29	146	69.52	205	97.16	159	75.71	175	83.33	1020	80.89

*BCG* Bacillus Calmette-Guerin, *Penta* pentavalent vaccine (vaccine against diphtheria, Tetanus, pertussis, hepatitis B and haemophilus influenzae), *OPV* oral polio vaccine, *Pneumo* Pneumococcus conjugated vaccine. Rota: Rotavirus vaccine

### Immunization status of children aged 12–23 months

In the enrolled households, 912 children were fully immunized, yielding a complete immunization coverage of 72.32% (95% CI 69.76–74.77), irrespective of the availability of immunization cards. Among the 1076 children with immunization cards, 872 (81.04%) were fully immunized. Among those who did not have an immunization card, 77.42 and 41.18% of the children were partially immunized in Maritime and Lomé HR respectively. Despite the absence of an immunization card, it was found that 86.67% of children in the Centrale-HR and 52.94% of children in the Lomé-HR had received all their vaccines.

### Barriers to immunization in the health regions

Main barriers to children’s immunization reported by respondents were: long distance to the health center (32.28%), poor road conditions (13.32%), lack of means of transport (10.55%) and lack of time (9.04%). The regional analysis shows that in the Lomé-HR, the main barrier was the lack of time (23.22%) while in the other regions, the remoteness of the health center was the main reason mentioned.

### Univariate and multilevel analysis

Table 3 summarizes univariate analysis. Four factors were associated with incomplete immunization: Monthly income (*p* = 0.035), marital status (*p* = 0.008), knowing that immunization is free of charge (*p* = 0.032), availability of immunization card (*p* < 0.001) and the health region (*p* < 0.001).

**Table 3:** Child immunization status at different levels of independent variables

Variables	Fully immunized		Not fully immunized		*P* values
	n	%	n	%	
Education level (*n* = 1173)					0.090
No education	323	71.8	127	28.2	
Up to primary	335	76.0	106	24.0	
Secondary and higher	222	78.7	60	21.3	
Age in years (*n* = 1189)					0.678
< 15	7	77.8	2	22.2	
15–49	866	75.2	286	24.8	
≥ 50	19	67.9	9	32.1	
Monthly income (*n* = 1206)					0.035
< 30,000 FCFA	851	74.6	289	25.4	
≥ 30,000 FCFA	57	86.4	9	13.6	
Walking time to reach health care center *n* = 1212)					0.229
< 30 min	341	73.8	121	26.2	
30–60 min	303	73.7	108	26.3	
> 60 min	266	78.5	73	21.5	
Marital status (*n* = 1208)					0.008
Married or living with partner	868	76.1	272	23.9	
Others	42	61.8	26	38.2	
Occupation (*n* = 1161)					0.095
Not working	32	66.7	16	33.3	
Worker	19	79.2	5	20.8	
Farming	331	71.8	130	28.2	
Income-generating activities	320	77.9	91	22.1	
Others	171	78.8	46	21.2	
Knowing that immunization is free of charge (n = 1208)					0.032
Yes	872	75.5	283	24.5	
No	33	62.3	20	37.7	
Place of vaccination (*n* = 1187)					0.374
Health care center	888	75.2	293	24.8	
Other	24	68.6	11	31.4	
Availability of immunization card (*n* = 1200)					< 0.001
Yes	872	82.1	190	17.9	
No	29	21.0	109	79.0	
Health region (*n* = 1216)					< 0.001
Lomé	179	85.2	31	14.8	
Maritime	133	65.5	70	34.5	
Plateaux	88	42.1	121	57.9	
Centrale	195	94.7	11	5.3	
Kara	150	73.2	55	26.8	
Savanes	167	91.3	16	8.7	

Multilevel multivariate logistic regression analyses results are presented in Table 4. The empty model (Model 1) showed that there was significant variability in the probability of children with incomplete immunization across districts [variance = 1.07, *p* < 0.001]. The inter-class correlation (ICC), 59.37% of variability in the probability of a child to have incomplete immunization was related to district-level factors. The variation in incomplete immunization in the other models (Model 2, Model 3 and Model 4) remained significant. In the Model 2, 62% of the variability was attributed to district-level factors. In addition, the proportional change in variance (PCV) for individual-level factors was estimated at 8.41% which means that, in the model, 8.41% of the variability in the probability of a child to have incomplete immunization across districts was explained by individual-level factors. With the full model (Model 4), we found that if a parent moves to another district that has a higher probability of incomplete child immunization, the likelihood of having his child not completely immunized would increase by 2.78 (median odds ratios (MOR) for the districts was 2.78). In the model 4, after controlling for both individual and contextual level variables, children whose parents or guardians attended secondary school or above were 33% (adjusted Odds Ratio (aOR) = 0.67, CI [0.47–0.94]) less likely to have an incomplete immunization than their counterparts with no educational background. The likelihood of incomplete immunization in children in this study decreased with increasing household’s income (aOR = 0.73, CI [0.58–0.93]). Conversely, children who did not have an immunization card (aOR = 13.41, CI [9.19–19.57]) and those whose parents did not know that children immunization is free of charge (aOR = 1.82, CI [1.00–3.30]) were more likely to have an incomplete immunization. In addition, children whose parents or guardians walked between half an hour and 1 h to go to the health center were 57% (aOR = 1.57, CI [1.15–2.13]) more likely to have an incomplete immunization compared to those whose guardians have to walk less than half an hour.

**Table 4:** Factors associated with incomplete immunization identified by multilevel multivariate logistic regression models

	Model 1 (empty) aOR [95% CI]	Model 2 aOR [95% CI]	Model 3 aOR [95% CI]	Model 4 aOR [95% CI]
*Individual factors*				
Education level				
No education		1		1
Primary school		0.75 [0.55–1.01]		0.76 [0.56–1.02]
Secondary or higher education		0.67 [0.47–0.94]		0.67 [0.47–0.94]
Monthly income *CFA/month*				
< 30,000 FCFA		1		1
≥ 30,000 FCFA		0.73 [0.57–0.94]		0.73 [0.58–0.93]
Having an immunization card				
Yes		1		1
No		13.35 [9.17–19.43]		13.41 [9.19–19.57]
Knowing that immunization is free of charge				
Yes		1		1
No		1.80 [1.03–3.40]		1.82 [1.003–3.30]
*Contextual factors*				
Walking time to reach a healthcare center				
Half an hour			1	1
Half an hour – 1 h			1.52 [1.09–2.12]	1.57 [1.15–2.13]
≥ 1 h			0.93 [0.64–1.35]	0.95 [0.67–1.36]
Health region				
Lomé			1.64 [0.51–5.33]	
Maritime			7.08 [2.46–20.37]	
Plateaux			16.72 [6.03–46.36]	
Centrale			1	
Kara			5.29 [1.80–15.51]	
Savanes			3.08 [1.05–8.98]	
Random effects				
District variance (SE)	1.07*(0.26)	1.16*(0.29)	0.53*(0.16)	1.16*(0.29)
ICC (%)	59.37	62.33	36.13	62.56
PCV (%)	Ref	−8.41%	50.5%	−8.41%
MOR	2.67	2.78	2.00	2.78
Model fit AIC	5243.54	5131.53	5890.03	5128.19

*aOR* ajusted Odds-Ratio, *SE* Standard Error, *AIC* Akaike Information Criterion, *CI* confidence interval, *ICC* intra-class correlation, *MOR* median odds-ratio, *PCV* proportional change in variance

Model 1 is the empty model, a baseline model with no independent variable

Model 2 is adjusted for individual factors (education, income, having immunization card and knowing that immunization is free)

Model 3 is adjusted for contextual factors (walking time, health region)

Model 4 is adjusted for individual and contextual factors

## Discussion

This nationwide survey that covered 1261 households from all the six health regions of Togo reported a vaccination coverage of 72.32% and also described factors associated with incomplete immunization coverage among children aged 12–23 months in 2017.

Immunization coverage has improved compared to national indicators observed in 2010 and 2013 with 63.8 and 61% respectively [12, 13]. This is one of the highest coverage reported in the West Africa region [15]. However, like in previous studies conducted in Togo and in other African regions, reported immunization coverage rates remain below the national target rate of at least 90% set by the WHO [2]. For example, a study conducted in the Western region of Cameroon in 2013 reported that 85.9% of children were fully vaccinated [16]. The antigen vaccination coverage for BCG was comparable to that of Cameroon in 2012 with 99.8%, and for the first and third doses of pentavalent with 98.9 and 94.8%, respectively [16]. In one regional State in Ethiopia in 2011, based on immunization card and recall, only three in ten (35.4%) children were fully vaccinated and 23.7% were unvaccinated [17] . In Nigeria, based on DHS data, only 23.7% of 5754 children aged 12–23 months were completely immunized [18]. In Senegal, the complete immunization rate was 62.8% according to the 2010–2011 DHS [10].

Several factors associated with incomplete vaccination have been identified: respondent’s educational level, household income, possession of immunization card, walking distance to healthcare center, and knowledge that immunization is free of charge. Similar factors have been found in the analyses conducted in 24 countries in sub-Saharan Africa with the enrolment of 27,094 children aged 12–23 months [19]. The first factor limiting the immunization coverage was the educational level of the respondents. In fact, it has been reported in many studies including ours that vaccination coverage was lower in children whose parents have a low level of education in Senegal [10], in Nigeria [20], in Kenya [21] and in Malawi [22]. Education has been reported to have profound effects on mothers’ health seeking behaviors which includes child immunization [18]. On this point, awareness campaigns need to be reinforced by relying on villages or community initiatives [23]. The involvement of the father can also play a decisive role in increasing immunization coverage.

Vaccination coverage was assessed by immunization card and parent’s recall. Immunization cards were available for 85% of children included in this survey. Not having the immunization card could be endogenous of immunization status. For example, a parent not knowing that vaccination is free of charge may end up with both partial immunization and loss of the card.

The immunization card remains an essential tool to check immunization status. Apart from national surveys, it must be checked by pediatricians or any health worker at each visit to a healthcare facility. A vaccination coverage study conducted in 2010 in Togo showed a vaccination card possession rate of 77% [12]. This rate of immunization card was higher compared to other studies. It was 50.2% in Cameroon in 2013 [16] and 41.8% in Ethiopia in 2011 [17]. Not having a vaccination card was associated with incomplete vaccination as also reported in Senegal [10] and in Ghana [24]. As the strongest variable for not being immunized is not having an immunization card, there is a need to educate parents of children regarding the importance of keeping immunization card in order to assess immunization coverage. Improving immunization card retention is one of the key measures which could help towards accurate estimation of vaccination coverage and to inform strong health policies. Therefore, innovative approaches that have proved to improve immunization coverage, such as the use mobile phones [25] should be urgently implemented and evaluated. Currently, studies are being conducted in Côte d’Ivoire using online card with short message service as means to increase immunization coverage. However, these approaches have not been yet evaluated.

We also observed important disparities in immunization coverage among health regions. One health region in Togo (Plateaux) had the lowest coverage (less than 50% coverage) because of difficulties in accessing vaccination centers. These obstacles to accessing vaccination centers are essentially related to the geographic characteristics of the region which is predominantly covered by mountains. In our study, only the Centrale-HR recorded a complete coverage of more than 90%. This difference could be explained by socio-cultural factors and easier access to vaccine services compared with Plateaux-HR. The socio-cultural factors referred to are mainly false beliefs such as the fear of being sterilized especially for male children and also that vaccination can cause death.

The socio-economic situation of the households was associated with incomplete vaccination. Similar findings were reported in Nigeria [18] and in Kenya [26]. The poorer a household is, the more likely it is for children of that household to have incomplete immunizations. As already reported by Landoh in Togo in 2010 [12], economic conditions measured by monthly salary influence immunization coverage. This is probably due to the cost of transportation and indirect costs such as purchase of vaccine cards and medication for vaccine related care.

Difficulties encountered to reach healthcare facilities are major barriers to child immunization completion. Similar findings were reported in Nigeria [18]. In our study, laborious access to vaccine services was measured by the walking time needed to reach the health care center from the house. This could be explained by poor road conditions or lack of vehicles to reach healthcare facilities. Most of the time, parents have to walk to reach the vaccination centers. Strengthening outreach strategies is important in the African context to improve immunization coverage.

The main bias for this survey was the use of parent’s recall to document immunization status. This could overestimate or underestimate the immunization coverage especially among children whose parents had lost the immunization card. The main difficulty encountered is the loss of vaccination cards for children in the country. It is therefore important to propose alternative vaccination registration models with the gradual introduction of new technologies in the collection of immunization data among children. We did not collect data on health services such as place of delivery and history of antenatal consultation, factors known to be associated with complete immunization coverage [18]. Despite this limitation, our findings are important to understand factors associated with immunization completion among children in Togo.

Compared to another study conducted in Togo, we used multilevel logistic regression analysis to identify risk factors for incomplete immunization status as conducted in a similar survey in Cameroun [16] and Nigeria [18]. Our study was population based and covered all six health regions in the country, hence yielding the results from this study to be generalized to the country.

## Conclusion

Our study revealed moderate vaccination coverage in Togo, although with high variation between regions. In the Plateaux-HR where access is difficult, the immunization coverage is less than 50%. Younger parents and families living away from the vaccination centers should be targeted with appropriate immunization promotion strategies. Information and attitude towards immunization should be strengthened. Interventions to improve child immunization uptake should be taken into account at the individual and community levels. These factors should be considered during the elaboration and implementation of national policies.

## Abbreviations

95%CI: 95% confidence interval
aOR: adjusted odds ratio
BCG: Tuberculosis vaccine
DHS: Demographic and Health Survey
DTP-3: Diphtheria-tetanus-pertussis
EPI: Expanded Program on Immunization
HR: Health region
ICC: Inter-class correlation
MICS4: Fourth multiple indicator cluster survey
MOR: Median odds ratios
OPV: Oral polio vaccine
PCV: Proportional change in variance
PENTA: Pentavalent vaccine
PNEUMO: Pneumococcal vaccine
ROTA: Vaccine against rotavirus gastroenteritis
WHO: World Health Organization
